# Response to ‘Increasing value and reducing waste in data extraction for systematic reviews: tracking data in data extraction forms’

**DOI:** 10.1186/s13643-018-0677-x

**Published:** 2018-01-25

**Authors:** Jens Jap, Ian J. Saldanha, Bryant T. Smith, Joseph Lau, Tianjing Li

**Affiliations:** 1 Center of Evidence Synthesis in Health, Brown University School of Public Health, Providence, USA; 2 Department of Epidemiology, Johns Hopkins Bloomberg School of Public Health, Baltimore, USA

## Abstract

This is a response to a Letter. Data abstraction is a time-consuming and error-prone systematic review task. Shokraneh and Adams categorize available techniques for tracking data during data abstraction into three methods: simple annotation, descriptive addressing, and Cartesian coordinate system. While we agree with the categorization of the techniques, we disagree with the authors’ statement that descriptive addressing is a PDF-independent method, i.e., any sort of descriptive addressing must reference a specific version of PDF file and not just any PDF of said report. Different versions of PDFs of the same report might place text and tables on different locations of the same page and/or on different pages. Consequently, it is our opinion that any kind of source location information should be accompanied by the source or linked by an intermediary service such as the Data Abstraction Assistant (DAA).

We read with great interest Shokraneh and Adams’ letter pertaining to data abstraction (or “data extraction”) during systematic reviews [1]. We agree with the authors that data abstraction is perhaps the most time-consuming task during systematic reviews and one that is error-prone [2–5]. Manual data abstraction, which is largely the current norm in the systematic review enterprise, is likely not sustainable in the long run. Software tools that help tracking of data to published reports of studies (i.e., PDFs) have the potential to greatly reduce the time spent and errors inherent to the data abstraction process [6]. We developed Data Abstraction Assistant (DAA), a software tool with this potential. By recording the exact location and mapping this location to the data entered into extraction forms, DAA could reduce errors and time spent reviewing extracted data [6].

Shokraneh and Adams categorize available techniques for tracking data into three methods: simple annotation, descriptive addressing, and Cartesian coordinate system [1]. The authors describe the second method, i.e., descriptive addressing, as one where the data abstractor abstracts data and notes each data point’s source of information (“address”) in the PDF, using the page, paragraph, line, table, figure, box, and/or headline numbers.

While we agree with the categorization of the techniques, we disagree with the authors’ statement that descriptive addressing is “PDF-independent.” In our experience, descriptive addressing indeed is dependent on the version of the PDF used; different versions of PDFs of the same report might place text and tables on different locations of the same page and/or on different pages. Consequently, it is our opinion that any kind of source location information should be accompanied by the source or linked by an intermediary service such as DAA. We developed DAA to facilitate data tracking between data abstraction forms and PDFs, thereby possibly reducing errors and saving time [6]. DAA allows users to mark and record the exact location of information found on a PDF. The locations are linked to data elements on a data extraction form. DAA enables users to create a link between information extracted and its source location. We are currently analyzing the results of a randomized controlled trial that formally evaluates the effectiveness of DAA (compared with standard data abstraction approaches) in improving these outcomes (i.e., error rates and time).

The use of DAA would not solve the challenge that copyright poses in sharing PDFs. However, by serving as an intermediary, linking the abstracted data and the exact location in the PDF source, DAA facilitates the efficient tracking of abstracted data.
